# Oral health-related quality of life of children with oral clefts and their families

**DOI:** 10.1590/1678-7757-2017-0106

**Published:** 2018-01-16

**Authors:** Gabriela Mendonça Rando, Paula Karine Jorge, Luciana Lourenço Ribeiro Vitor, Cleide Felício Carvalho Carrara, Simone Soares, Thiago Cruvinel Silva, Daniela Rios, Maria Aparecida Andrade Moreira Machado, Maria Beatriz Gavião, Thais Marchini Oliveira

**Affiliations:** 1 Universidade de São Paulo, Faculdade de Odontologia de Bauru, Bauru, SP, Brasil.; 2 Universidade de São Paulo, Faculdade de Odontologia de Bauru, Departamento de Odontopediatria, Ortodontia e Saúde Coletiva, Bauru, SP, Brasil.; 3 Universidade de São Paulo, Faculdade de Odontologia de Bauru, Departamento de Odontopediatria, Ortodontia e Saúde Coletiva; Hospital de Reabilitação de Anomalias Craniofaciais, Bauru, SP, Brasil.; 4 Universidade de São Paulo, Faculdade de Odontologia de Bauru; Hospital de Reabilitação de Anomalias Craniofaciais, Departamento de Prótese, Bauru, SP, Brasil.; 5 Universidade Estadual de Campinas, Faculdade de Odontologia de Piracicaba, Departamento de Odontopediatria, Piracicaba, SP, Brasil.

**Keywords:** Quality of life, Oral health, Questionnaires, Cleft lip, Cleft palate

## Abstract

Oral health problems can influence people's Quality of Life (QoL) because of pain, discomfort, limitations, and other esthetics problems, affecting their social life, feeding, daily activities, and the individual's well-being.

**Objective:**

To compare oral health-related quality of life (OHRQoL) of children with and without oral clefts and their families.

**Materials and Methods:**

121 children aged from 2 to 6 years, from both sexes, enrolled in the treatment routine of the Pediatric Dentistry Clinics of a Dental School and a Hospital for Cleft Treatment were divided into two groups: Group 1 - children with cleft lip and palate; Group 2 - children without cleft lip and palate. The OHRQoL was assessed using the validated Portuguese version of the *Early Childhood Oral Health Impact Scale* (B-ECOHIS). The questionnaire was answered individually, only once, at a private place. Mann-Whitney U test was used to verify differences between groups. Spearman's Rho test was used to associate sex and age with quality of life. The level of significance was set at 5% (p<0.05).

**Results:**

According to the parents’ perception on the OHRQoL of children with and without cleft lip and palate, oral health of children with oral clefts (Group 1) had a statistically significant impact on OHRQoL. The correlation of sex with impact on OHRQoL did not show statistically significant differences. On the other hand, the higher the age the higher the impact on QoL.

**Conclusions:**

The group comparison revealed that the cleft lip and palate negatively impacted on OHRQoL of 2 to 6-year-old children and their parents.

## Introduction

The relationship between quality of life (QoL) and oral health has gained attention in Dentistry because of the importance of oral health problems resulting in physical and psychosocial impacts on people's lives. Oral health problems can cause pain, discomfort, and put on some limitations, and other esthetics problems that affect the individual's social life, feeding, daily activities, and well-being, consequently leading to significant impacts on QoL^18^. Thus, it is important to understand how a person understands the oral condition, because the behavior is conditioned by this perception.

The oral health of children affects feeding, smiling, speaking, and socialization. The facial appearance and its relation with body image, self-stem, and emotional well-being play an important role in social interaction. Thus, interfering in these functions will influence the QoL of these children. Negative feelings regarding facial esthetics make the child believe that oral health negatively affects their daily life activities^24^. The oral health-related quality of life (OHRQoL) is an important auxiliary measure for clinical indicators to assess health, especially in children. Many studies proved the impact of oral alterations on QoL of children of different ages and their families^1^ ^,^ ^12^ ^,^ ^17^ ^,^ ^26^ ^,^ ^27^.

Because of the increasing understanding of QoL as an important measure for dental treatment, specific tools to measure the influence of oral alterations in daily activities are necessary^28^. These tools also show the psychosocial impact of the main oral alterations on QoL of children of different ages^1^ ^,^ ^17^ ^,^ ^20^ ^,^ ^26^ ^,^ ^27^. In dentistry, usually children undergo interviews or fill in questionnaires on how oral problems have impact on daily activities^1^ ^,^ ^4^ ^,^ ^13^ ^,^ ^15^ ^,^ ^23^.

Considering traditional clinical indicators can evaluate the pathology, but not its effects, especially on children, QoL of children is an auxiliary tool to measure health^6^.

Cleft lip and palate is a morphological alteration that causes esthetic and functional problems with psychosocial implications in the individual's life and well-being^11^. Thus, cleft lip and palate rehabilitation primarily aims at fully integrating the individual in society^10^ ^,^ ^11^. This rehabilitation treatment starts in the childhood with primary plastic surgeries to repair the cleft lip at 3 months of age and the cleft palate at 12 months, and only finishes at adulthood, lasting for the individual's entire life^11^. Knowledge on OHRQoL improves treatment quality and is of extreme importance in the rehabilitation process of children with oral clefts that comprises multidisciplinary care aiming at satisfactory QoL^26^. Notwithstanding, studies on QoL of children with cleft lip and palate and their relatives are still scarce^2^ ^,^ ^5^ ^,^ ^14^.

This study evaluated the OHRQoL of 2-6 year-old children with and without cleft lip and palate and their relatives with a validated *Early Childhood Oral Health Impact Scale* (B-ECOHIS) questionnaire (Portuguese version)^6^ ^,^ ^19^ ^,^ ^21^ ^,^ ^25^ ^,^ ^30^.

## Material and methods

### Participants

This study was submitted and approved by the Institutional Review Board according to the ethical issues (protocol no. CAAE #41274215.9.0000.5441). All parents/legal guardians were instructed on the research and signed a free and clarified consent form.

### Interview

The inclusion criteria were: children aged from 2 to 6 years, from both sexes, enrolled in the routine treatment of the Pediatric Dentistry Clinic of a Dental Institution and a Hospital for Specialized Cleft Care. Exclusion criterion was the presence of syndromes or other anomalies. The selected children were divided into two groups: Group 1 - children with cleft lip and palate (n = 75) and Group 2 - children without cleft lip and palate (n=46).

The children's OHRQoL was assessed by applying the questionnaire (B-ECOHIS - Portuguese version)^6^ ^,^ ^19^ ^,^ ^21^ ^,^ ^25^ ^,^ ^30^ with the parents of the children. The QoL was evaluated using a questionnaire answered by the parents on the OHRQoL of 2 to 5-year-old children (ECOHIS)^21^ ^,^ ^30^. The ECOHIS questionnaire comprised 13 multiple-choice questions: 9 questions evaluated the impact of oral problems on the child and 4 questions evaluated the impact of oral problems on the child's family^21^ ^,^ ^30^. The parents’ answers were categorized as it follows: 0=never; 1 = almost never; 2=sometimes (on occasion); 3=frequently; 4=very frequently; 5 = I do not know.

For each child, a global impact score was obtained by summing the scores (from zero to four) of the 13 questions^21^ ^,^ ^30^. The questionnaires with two or more questions answered with “I do not know” were excluded from the analyses. The OHRQoL impact was classified with “without impact” (for the answers “never” and “almost never”) and “with impact” (for the answers “sometimes”, “frequently” and “very frequently”)^1^ ^,^ ^21^. Questionnaires with at least one question answered with “sometimes”, “frequently” and “very frequently” either in the child or family domains were considered of negative impact on OHRQoL of either the child or the family, respectively.

The questionnaire was individually answered by the parents, at a private place of the Baby Clinics of the Dental Section of the Hospital for the Rehabilitation of Craniofacial Anomalies (HRAC/USP) or the School of Dentistry of Bauru/USP (FOB/USP). The participant's confidentiality and privacy were assured. It took approximately 10 minutes to answer the questionnaire. If oral problems were detected, the child was referred to evaluation in the Pediatric Dentistry Clinics of HRAC/USP or FOB/USP.

### Statistical analysis

Data were analyzed using the Statistical Package for the Social Sciences software (SPSS_version 21) (IBM, Armonk, NY, USA). The reliability of the answers was confirmed by Cronbach's alpha. This coefficient is extensively used in QoL research that use questionnaires. The Cramer's V test was used to analyze the statistical differences in the variable distribution in three or more categories between groups. The Mann-Whitney's U test was used to verify statistical differences between groups. The Spearman's Rho test was used to correlate sex and age with QoL, at a significance level of 5% (p<0.05).

## Results

From 150 questionnaires, we selected 121 to comprise the sample according to the inclusion criteria, because some questionnaires were not completely filled in, thus being excluded from the sample.

We confirmed the reliability of the parent's answers using Cronbach's alpha, which revealed coherence in answers to the questionnaire, according to the variance of each item and the coefficient's result (Table 1). The normal distribution test (Kolmogorov-Smirnov; *p*<0.0001) rejected the normality hypothesis.

**Table 1:** Answers internal consistency according to the variance of each item and to Cronbach's alpha

	Cronbach's alpha	ICC (95% CI)
Impact on the child	0.77	0.77 (0.70-0.83)
Impact on the family	0.60	0.60 (0.48-0.70)
ECOHIS - Total	0.80	0.80 (0.75-0.85)

The descriptive analysis of the results (Table 2) shows the questions on the impact of OHRQoL on the child's life (numbered from 1 to 9) that address tooth pain, and the difficult in eating certain foods, drinking hot or cold beverages, speaking, going to school, and performing daily life activities. The questions on the impact of OHRQoL on the family's life (numbered from 10 to 13), addressed the feeling of anger and guilt for missing days at work because of the treatment, and the financial impact of dental treatment on the family.

**Table 2:** Answers distributions to the questions 1-13 regarding the domains - impact on the child and on the family

Questions		Group 1			Group 2		Cramer's V	p
	Never, almost never n(%)	Sometimes, frequent, very frequent n(%)	I don't know n(%)	Never, almost never n(%)	Sometimes, frequent, very frequent n(%)	I don't know n(%)		
Impact on the child								
1	55 (45.4%)	16 (13.2%)	4 (3.3%)	44 (36.3%)	2 (1.65%)	0 (0%)	0.283	0.008*
2	62 (51.2%)	11 (9.09%)	2(1.65%)	44 (36.3%)	2(1.65%)	0 (0%)	0.195	0.100
3	57 (47.1%)	17 (14.04%)	1 (0.82%)	42 (34.71%)	2(1.65%)	2(1.65%)	0.256	0.019*
4	52 (42.9%)	19 (15.7%)	4 (3.3%)	42 (34.71%)	2(1.65%)	2(1.65%)	0.274	0.011*
5	61 (50.4%)	14 (11.5%)	0 (0%)	40 (33%)	5 (4.13%)	1 (0.82%)	0.153	0.241
6	67 (55.3%)	7 (5.78%)	1 (0.82%)	44 (36.3%)	1 (0.82%)	1 (0.82%)	0.142	0.293
7	59 (48.7%)	13 (10.7%)	3 (2.47%)	43 (35.5%)	2 (1.65%)	1 (0.82%)	0.201	0.086
8	69 (57.02%)	6 (4.95%)	0 (0%)	45 (37.1%)	1 (0.82%)	0 (0%)	0.121	0.183
9	68 (56.1%)	6 (4.95%)	1 (0.82%)	46 (100%)	0 (0%)	0 (0%)	0.194	0.102
Impact on the family								
10	58 (47.9%)	16 (13.2%)	1 (0.82%)	42 (34.71%)	4 (3.3%)	0 (0%)	0.183	0.133
11	60 (49.5%)	12 (9.91%)	3 (2.47%)	38 (31.4%)	7 (5.78%)	1 (0.82%)	0.052	0.851
12	55 (45.4%)	20 (16.5%)	0 (0%)	41 (33.8%)	5 (4.13%)	0 (0%)	0.189	0.037*
13	64 (52.8%)	11 (9.09%)	0 (0%)	45 (37.1%)	0 (0%)	1 (0.82%)	0.271	0.012*

* statistically significant difference

According to the parents’ perception on the OHRQoL of children with and without clefts, the statistical analysis of the questionnaire showed a statistically significant difference between groups with higher impact of the cleft on the OHRQoL (Table 3).

**Table 3:** Comparison between Groups 1 and 2 - Mann-Whitney U test

	Mean±SD	Median	Mean Rank	Mann-Whitney
Group 1	6.25±6.60	4.00	68.09	1.193
Group 2	2.98±5.56	1.00	49.45	p=0.003*

* statistically significant difference

The analysis of the correlation between sex and impacts on QoL did not show statistically significant differences.

The age of the studied children ranged from 24 to 72 months (average of 46.45 months). The analysis of the correlation between age and impacts on QoL revealed that the higher the age, the higher the impact on QoL (Table 4).

**Table 4:** Correlation between sex/age and the impact on QoL (Spearman's Rho test)

	Spearman's Rho	Impact on QoL
Sex	-0.009	p = 0.953
Age	0.323	p = 0.029*

* statistically significant difference

## Discussion

Recent studies demonstrate that reports of children on OHRQoL are reliable and valid. Instruments developed to measure OHRQoL of children should also assess the impact of these problems on the family's QoL, because they are inseparable factors^12^ ^,^ ^17^ ^,^ ^23^ ^,^ ^26^ ^,^ ^27^ ^,^ ^31^. The assessment of OHRQoL of the child reflects on the parents’ perception towards their own oral health, thus improving the communication between children, parents, and dental heath professionals^33^. Awoyale, et al.^5^ (2015) evaluated the factors affecting the QoL of families of children with cleft lip and palate. The authors stated that to improve the QoL of these families, there is need for scheduling individual counseling after the child's birth, which would contribute to a better understanding of the consequences of oral health on the child's and family's lives, to care prioritization and to the consequence estimation of treatment strategies and initiatives^6^.

The QoL measurement of children involves methodological problems such as: the perception of children of different ages; difficult in separating the parents’ perception from the child's perception; and the range in the number of activities according to age. However, with the correct translation and application of techniques, the B-ECOHIS is an appropriate and reliable method to assess the QoL of elementary students. The perception of the parents on their children's oral health is important for the dentists’ knowledge on providing proper care. Thus, there is need to correlate the perceptions of the adult, the child, and the family, which can either unable or enable the access to dental care^1^. Jokovic, et al.^13^ (2004) showed that the questionnaire is an effective tool to assess the perceptions on the impact of oral diseases on QoL. Barbosa, Vicentin and Gavião^6^ (2011) stated that the use of validated questionnaires is an innovative and promising proposal in Pediatric Dentistry because dentists still use tools designed for clinical practice that are mostly inappropriate in other health contexts. Shaghaghian, Bahmani and Amin^27^ (2015) found that oral health quality of elementary students had a significant impact on OHRQoL. These authors reported that health promotion strategies and the parents’ attitude regarding toothbrushing can positively influence the child's oral health, both being highly recommended.

The ECOHIS questionnaire has been widely used to assess the impact that several oral problems have on QoL^6^ ^,^ ^12^ ^,^ ^19^ ^,^ ^21^ ^,^ ^25^ ^,^ ^26^ ^,^ ^29^ ^,^ ^31^. Scarpelli, et al.^26^ (2013) evaluated the QoL of 5-year-old children from different social classes regarding the presence of early childhood caries, tooth trauma, malocclusion, the developmental enamel defects, and DTMF using ECOHIS associated with a socioeconomic questionnaire. Sousa, et al.^29^ (2014) applied B-ECOHIS to assess the impact of malocclusions on the QoL of preschoolers and their families considering the esthetic and functional consequences of this oral problem Gomes, et al.^12^ (2014), through ECOHIS, evaluated the QoL of 843 children aged between 3 to 5 regarding caries, tooth trauma, and malocclusion from the physical and psychological consequences of these conditions. Viegas, et al.^31^ (2014), using ECOHIS, studied the impact of tooth traumas on preschoolers and their families and found a positive relation for avulsed teeth. We chose this methodology to address not only the diagnosis and treatment of the cleft, but also the impacts on the QoL of the children and their families.

It is worth to highlight that OHRQoL of children with oral clefts has gained interest because oral disorders can affect QoL negatively^7^ ^,^ ^32^. Depending on the cleft type, children with cleft lip and palate are stigmatized because of facial appearance (cleft lip), speech (cleft palate), or both (cleft lip and palate); thus, they are at higher risk of developing functional, social, and emotional alterations during childhood^16^. In this sense, questionnaires aiming at assessing the impact of oral health on the people's well-being have been developed and adapted^2^ ^,^ ^3^ ^,^ ^7^ ^-^ ^9^ ^,^ ^16^ ^,^ ^22^ ^,^ ^23^ ^,^ ^32^. The OHRQoL of children with oral clefts was statistically different from that of children without oral clefts, *i.e.,* the QoL of children without clefts was higher than the QoL of children with clefts, corroborating with the findings by Antonarakis, Patel and Tompson^2^ (2013). These authors conducted a systematic review to evaluate oral health quality of non-syndromic individuals with cleft lip and palate compared to individuals without clefts, and observed that the former had poorer QoL than the latter. Antunes, et al.^3^ (2014) analyzed, using the B-FIS scale, the impact of OHRQoL on the families of children with non-syndromic oral clefts matched to children without oral clefts regarding age, sex, geographic distribution, and socioeconomic level. The children with oral clefts had a higher impact on QoL of the families, which corroborated with the findings of this study. It is important to emphasize that this study found statistically significant differences (Table 2), in the questions #1, #3, and #4 on the impact of oral health on QoL of the children with oral clefts. We also found statistically significant differences also occurred regarding the impact of oral health on QoL of the families (problems concerning missed days at work because of treatment and the financial impact of the dental treatment on the families of children with cleft lip and palate (questions #12 and #13).

Aravena, et al.^4^ (2015) compared the OHRQoL of children with and without clefts, aged from 8 to 15 years, using the COHIP-SP (Spanish translation). Compared to this study, the OHRQoL of children with and without oral clefts were similar, but the age range of the study was different. On the other hand, Kortelainen, et al.^15^ (2015) observed that the OHRQoL of Finnish children with cleft lip and palate was considerably worse than that of children without cleft lip and palate, which is similar to the findings of this study, although the used instrument was CPQ_11-14_ for children aged between 11 and14. In this study, the higher the age, the higher was the impact of oral health on QoL, probably because of the problems associated with speaking and other people's understanding^4^. Further studies are necessary to verify the methodologic differences^23^ and clarify these possible associations.

The literature lacks studies on validated and reliable instruments to understand the perception of OHRQoL of individuals with oral clefts. Further studies employing specific tools to assess QoL with larger samples are important to understand the QoL of children with oral clefts. Furthermore, future research should include questions and answers regarding OHRQoL because this information can contribute to a better understanding of the rehabilitation process and to achievea better QoL of children with cleft lip and palate.

## Conclusion

Based on the results obtained, we concluded that the group comparison revealed that the cleft lip and palate presence had a negative impacted on OHRQoL of children from 2 to 6 and their parents.
